# Management Outcome in Simple and Complex Hydatid Cysts of Lung

**DOI:** 10.7759/cureus.12212

**Published:** 2020-12-22

**Authors:** Pratikshya Thapaliya, Tanveer Ahmad, Ambreen Abid, Nazish Sikander, Misauq Mazcuri, Nadir Ali

**Affiliations:** 1 Thoracic Surgery, Jinnah Postgraduate Medical Centre, Karachi, PAK

**Keywords:** hydatid disease, zoonosis, simple hydatid cyst, complex hydatid cyst, management outcomes

## Abstract

Introduction: Hydatid cyst (HC) of lung is a frequently encountered entity in Pakistan. The clinical and radiological manifestations of HC in lung depend on the integrity of the cyst. Patients may remain asymptomatic for years in cases of simple HC or may present with a wide variety of complications when it ruptures. The aim of this study was to compare management outcomes in simple and complex HCs.

Methods: This prospective, observational study was conducted from February 2019 until May 2020. Patients were divided into two groups: simple HC (Group A) and complex HC (Group B). Preoperative complications, surgical procedures, postoperative complications, duration of hospital stay, duration of chest tube placement and need for readmission were noted and compared between the two groups. All data was processed through the Statistical Package for the Social Sciences (SPSS) Statistics version 22 (IBM Corp., Armonk, NY).

Results: Sixty-two patients were included out of which Group A had 28 (45.2%) patients and Group B had 34 (54.8%) patients. There were 39 (62.9%) males and 23 (37.1%) females. The mean age was 31.11 ± 11.02 years. Preoperative complications in Group B included empyema seen in 10 (28.5%) patients, rupture of cyst into bronchus in 8 (23.5%), biliopleural fistula in 4 (11.7%), hydropneumothorax in 2 (5.8%), bronchopleural fistula in 1 (2.9%), airway compromise in 1 (2.9%) and pneumonia in 1 (2.9%) patient. Group B required longer days of chest tube placement, longer intensive care unit stay and longer hospital stay (p<0.001). The frequency of postoperative intervention was more in group B (p<0.05), therefore requiring readmission.

Conclusion: Surgery has favorable outcomes in the management of HC of lung. Complications associated with complex HC not only requires preoperative intervention like chest tubes but can also lead to life-threatening complications. There is also a frequent need for additional procedures during surgery in cases with complex HC along with greater risk of postoperative complications. All these are associated with prolonged hospital stay, readmissions and greater morbidity. Hence, early diagnosis and referral is needed to avoid these preventable complications associated with cyst rupture.

## Introduction

Hydatid disease is a zoonotic infection caused by the parasite *Echinococcus granulosus*[1,2]. It is a disease of public health concern and is endemic in regions involved in animal farming worldwide including Pakistan [3,4]. A large proportion of Pakistani population consists of professional farmers involved in agriculture and animal husbandry and most of them are living under poor hygiene and sanitary conditions [2-5]. In the life cycle of the parasite, dogs and carnivores are definite hosts while sheep and cattle are intermediate hosts^ ^[5]. Human is an accidental host infected through contact with the definitive host or by consuming water or vegetables contaminated with dog feces[5,6].

In this disease, liver is the most common organ involved (63%) followed by lungs (25%)[1]. A hydatid cyst (HC) has the ability to grow in lungs due to its elasticity and compliance and can range from 1 to 15 cm in size[2-4]. Cysts may remain asymptomatic for a long period and may only be symptomatic when they grow to a certain size or when complications arise [4,7,8]. Initial diagnosis can be made on the basis of history and chest X-ray along with positive serum echinococcal antibody [9]. However, a negative serologic test does not rule out the diagnosis of HC [2]. A computerized tomography (CT) scan is useful for confirming diagnosis and also helps to identify complications along with extra-pulmonary and extra-thoracic location of cysts. A ruptured cyst shows characteristic changes in radiographs as a result of air entering the cyst and separating the cyst layers from the surrounding host tissue [10]. This gives rise to certain diagnostic signs that include water lily sign, crescent and inverse crescent signs and ring enhancement sign [10].

In Pakistan, hydatid disease is one of the most important neglected tropical diseases. According to a report, 270 million people (58%) of the total population are at risk of hydatid disease in Central Asia, including Pakistan [3]. Therefore, more research and public awareness is important to reduce the burden of this disease [3]. HC of lung is frequently encountered in the daily practice of thoracic surgeons in Pakistan. We conducted this study to compare simple and complex HCs and their management in our setup. To our knowledge, no similar comparative study has been conducted locally so far. We believe this article will give some insight to chest care physicians and surgeons regarding the importance of early intervention to prevent morbidity in hydatid lung disease.

## Materials and methods

This prospective, observational study was conducted in the Department of Thoracic Surgery, Jinnah Postgraduate Medical Center, Karachi, from February 2019 till May 2020. Informed consent from patients was obtained and the study was approved by the Institutional Review Board (IRB no. F.2-81/2019-GENL/11632/JPMC, dated Feb 15, 2019).

**Figure 1:**
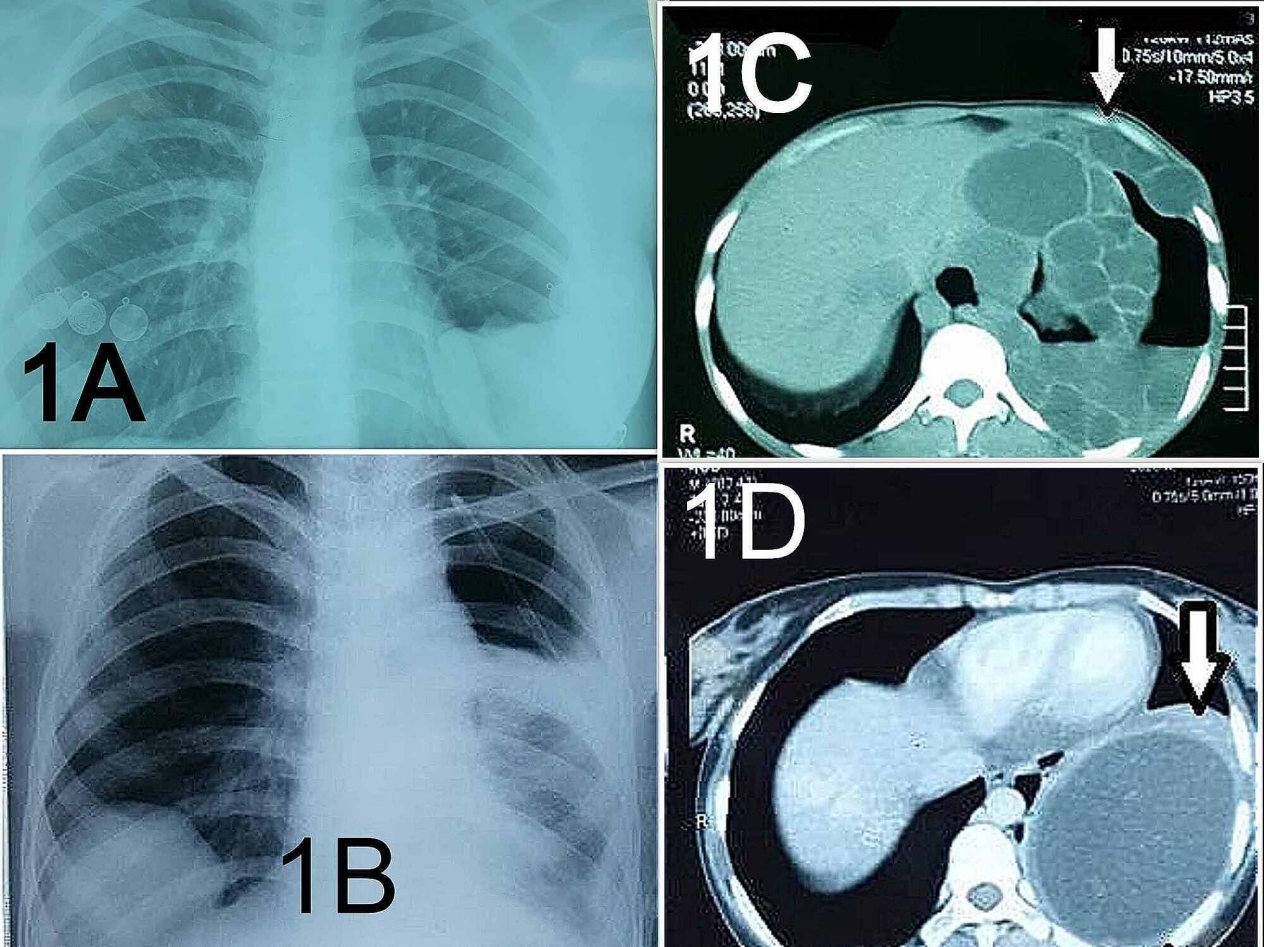
Chest X-rays and CT scans of HC in the lung (1A) Chest X-ray showing a ruptured HC in the left lung with water lily sign. (1B) Chest X-ray showing a simple HC on the right and hydropneumothorax with a chest tube on the left after the rupture of HC. (1C) CT scan of the complex HC with multiple daughter cysts in the left hemithorax extending into the left chest wall. (1D) CT scan of a simple HC in the left lung. HC, hydatid cyst.

All patients above 12 years of age presenting with clinical and radiological signs of HC of lung were included. Patients unfit for surgery or in whom spontaneous resolution occurred after rupture were excluded. Diagnosis was made with history, clinical examination, positive serum echinococcal antibody along with findings on chest X-ray and CT scan of the chest and abdomen. We did not refute diagnosis solely on the basis of a negative serum echinococcal antibody. Patients were divided into two groups. Group A included simple HC and group B included complex HC. Simple HC is defined as a well-defined cyst in the pulmonary field without signs of rupture. Complex HC is defined as the cyst that has ruptured into the bronchus or pleural cavity with or without infection [11]. Radiological signs of rupture were presence of air crescent, air fluid level within the cyst, water lily sign and ring enhancement sign [10]. Complications such as pneumothorax, pleural effusion, hydropneumothorax, empyema leading to entrapped lungs and consolidations were noted on the radiograph. Different radiological presentations of HC are shown in Figure 1. 

All patients were treated surgically after informed consent by a single thoracic surgeon in our setup. All patients were operated on single lung ventilation with double-lumen endotracheal tubes. Standard posterolateral thoracotomy incision was utilized. In Group A, the surgical wound and pleural cavity were packed with wet packs soaked either in 10% povidone-iodine solution or 10% hypertonic saline to prevent contamination. Cystostomy, removal of the germinal layer and bronchial leak closure were performed in all cases. Capitonnage was done to obliterate the cavity using polyglactin (Vicryl®) 3/0 sutures. Complete closure of the cystic space was not carried out in severely infected cases.

Additional surgical procedures required were noted. Decortication was performed for entrapped lungs due to empyema, and phrenotomy and repair were done for transdiaphragmatic communication of the hepatic cyst into the pleural cavity. Non-anatomic lung resection was done when there was unsalvageable parenchymal destruction or involvement of almost the whole lobe by the cyst. At the end of the procedure, air leaks were checked under normal saline with positive pressure ventilation of 30-40 cm H_2_O and secured. A single 32-Fr chest tube was placed before the closure of chest.

All patients received perioperative broad-spectrum antibiotics prophylactically with adequate postoperative analgesia and chest physiotherapy. The chest tube was removed when no air leak was evident, when drainage was less than 100 ml per day and radiologically the lung was expanded. Prolonged air leak was defined as postoperative air leak persisting for greater than seven days. A persistent air leak for more than 14 days was labeled as bronchopleural fistula (BPF). After discharge, the follow-up regimen consisted of chest X-ray on the seventh day and then biweekly for one month. Postoperatively all patients were given albendazole therapy for three months with a dose of 10 mg/kg/day.

Clinical information, preoperative complications, preoperative interventions, operative procedures, postoperative complications, duration of air leak, duration of intensive care unit (ICU) stay, duration of hospital stay and readmission were recorded in a predesigned pro forma. All data was processed through the Statistical Package for the Social Sciences (SPSS) Statistics version 22 (IBM Corp., Armonk, NY). Descriptive statistics were presented as means and standard deviations (SDs). Categorical variables were presented as frequencies and percentages. Chi- square test and independent t test were used to find association between categorical data and continuous data wherever appropriate. A p value ≤0.05 was taken as statistically significant.

## Results

A total of 62 patients were included in this study; 23 (37.1%) were females and 39 (62.9%) were males. The mean age was 31.11 ± 11.02 years. Majority of the patients were from Sindh, 34 (54.8%); followed by Baluchistan, 18 (29.0%); Afghanistan, 7 (11.3%) and Punjab, 3 (4.8%). History of animal contact was positive among 38 (61.3%) patients. Group A included 28 (45.2%) patients with simple HC and Group B included 34 (54.8%) patients with complex HC. The size of cyst in Group A ranged from 3 to 10 cm with a mean of 6.46 ± 1.8 cm. Chest pain was the most frequently observed symptom in both the groups. Fever, productive cough, dyspnea, hemoptysis and expectoration of germinal membrane were statistically significant among patients in Group B (p<0.001). A comparison of clinical manifestations among Groups A and B is given in Table 1.

**Table 1:** Comparison of clinical manifestations in Groups A and B

Clinical manifestations	Group A (simple hydatid cyst)	Group B (complex hydatid cyst)	p value
Chest pain	17 (60.7%)	26 (76.5%)	0.144
Cough	13 (46.4%)	13 (38.2%)	0.347
Dyspnea	6 (21.4%)	22 (64.7%)	0.001
Productive cough	3 (10.7%)	20 (58.8%)	<0.001
Fever	3 (10.7%)	20 (58.8%)	<0.001
Expectoration of germinal membrane	-	10 (29.4%)	0.008
Hemoptysis	-	14 (41.2%)	<0.001
Abdominal pain	4 (14.3%)	12 (35.3%)	0.054

Intrathoracic locations of HC were right lung 33 (53.2%), left lung 20 (32.2%) and bilateral 9 (14.5%). HC was most frequently observed in the lower lobe among 44 (70.9%) of patients. The extra-thoracic location of the cyst was noted in the liver in 37 (59.7%) patients. Peritoneum was also involved in 10 (16.1%) patients. History, chest X-ray and contrast CT scan helped to reach correct diagnosis in all the cases. Echinococcal antibody was positive among 40 (64.5%) patients. There was no significant correlation between the echinococcal antibody and type of cyst. Among Group B, the most frequent preoperative complication was empyema in 10 (28.57%), followed by rupture into bronchus in 8 (23.52%), biliopleural fistula in 4 (11.76%), hydropneumothorax in 2 (5.8%), bronchopleural fistula in 1 (2.94%), airway compromise in 1 (2.94%) and pneumonia in 1 (2.94%).

Preoperative interventions performed prior to definite surgery in cases with complex HCs were tube thoracostomy in 23 (67.6%) patients and bronchoscopy in 2 (5.88%) patients. Two diagnostic needle thoracentesis were performed in another center prior to referral. One of the patients required endotracheal intubation and ventilator support for massive air leak post-tube thoracostomy and had to undergo emergency surgery. One of the patients required emergency bronchoscopy for airway compromise due to the rupture of HC into bronchus. All other patients were operated electively. Patients of liver dome cyst with transdiaphragmatic extension into the thorax were operated in the same setting for liver cyst. Patients who had HC in the liver without thoracic communication were referred to a general surgeon for staged surgery. Additional surgical procedure required other than cystostomy along with bronchial leak closure and capitonnage were decortications (Group A=0, Group B=17; p<0.001), phrenotomy with diaphragmatic repair (Group A=0, Group B=12; p<0.001) and lung resection (Group A=2, Group B=12; p=0.008). A comparison of frequencies of postoperative complications among two groups is given in Table 2.

**Table 2:** Frequency of postoperative complications in Groups A and B

Post-operative complication	Group A (simple hydatid cyst)	Group B (complex hydatid cyst)	p value
Atelectasis	14 (36.8%)	24 (63.2%)	0.082
Partial lung expansion	1 (10.0%)	9 (90.0%)	0.015
Empyema	-	9 (100%)	0.003
Wound infection	2 (22.2%)	7 (77.8%)	0.128
Prolong air leak	-	8 (100%)	0.005
Loculated pneumothorax	1 (20.0%)	4 (80.0%)	0.244
Bronchopleural fistula	-	4 (100%)	0.083
Biliopleural fistula	-	3 (100%)	0.158
Pneumothorax	1 (33.3%)	2 (66.7%)	0.574

 Patients in Group B required longer days of chest tube placement, longer ICU stay and longer hospital stay when compared to Group A showing statistical significance (p<0.001). The frequency of postoperative intervention was higher in Group B (p<0.05). In Group A, 2 (7.1%) patients required readmission whereas in Group B, 12 (35.3%) patients needed readmission. The need for readmission was higher in Group B (p<0.05). A comparison of postoperative ICU stay, chest tube duration, duration of hospital stay and frequencies of postoperative intervention among two groups is summarized in Table 3.

**Table 3:** Postoperative characteristics of Groups A and B

Mean (SD)	Group A (simple hydatid cyst)	Group B (complex hydatid cyst)
ICU stay (days)	0.68 ± 0.905	2.21 ±1.737
Chest tube placement (days)	3.36 ± 1.162	10.76 ± 7.402
Hospital stay (days)	5.18 ± 1.657	10.71 ± 4.726
Postoperative intervention		
Tube thoracostomy	1 (3.6%)	5 (14.7%)
Thoracocentesis	-	5 (14.7%)
Pleurocutaneous window for open drainage of empyema	-	1 (2.9%)

One patient in Group A required tube thoracostomy for pneumothorax postoperatively. Both groups responded well to surgical management with no mortality.

## Discussion

To our knowledge, our hospital is the only tertiary care referral center providing Thoracic Surgery facility in the region, and most of our patients were referred from different areas of rural Sindh. We also received patients from Baluchistan and neighboring Afghanistan in significant numbers, which indicates the prevalence of the disease in these endemic areas. Clinical presentation depends on whether the cyst is intact or ruptured [4,11]. Rupture of the cyst into bronchus causes hemoptysis or expectoration of the cystic content [12-15]. Purulent sputum with fever might occur in cases with infected cyst. Dyspnea and bronchospasm might occur due to anaphylactic reaction to the hydatid antigen[2,11]. In our study, the most common presenting symptom among both groups was chest pain, which is similar to another study [11]. Fever, productive cough, hemoptysis and expectoration of germinal membrane were statistically significant among Group B (p<0.001). These findings were in line with a previous study by Kuzucu et al., who had reported that hemoptysis, sputum and fever are significantly more frequent in complex HC (p<0.05)[11]. Furthermore, pleural complications noted among Group B included empyema thoracis, pneumothorax, biliopleural fistula, hydropneumothorax and BPF. In addition to these, sometimes life-threatening complications might arise following the rupture of HC. Rupture of cyst may lead to asphyxia due to flooding of bronchial tree with hydatid fluid or blockage of bronchial lumen due to germinal membrane [12]. We had two cases where emergency intervention and surgery were needed. One patient had a massive air leak and subcutaneous emphysema following tube thoracostomy causing inability to maintain tidal volume on a ventilator requiring emergency thoracotomy. The other patient developed airway compromise due to rupture of cyst content into the bronchus for which urgent bronchoscopy followed by surgery was performed. Kilic et al. proposed that the larger size of the cyst (>10 cm) had a higher risk of spontaneous rupture [13]. We observed that the size of the cyst in Group A ranged from 3 to 10 cm with a mean of 6.46 ± 1.8 cm. We did not observe massive hemoptysis, tension pneumothorax and anaphylaxis.

There should be a high clinical suspicion when a patient from an endemic area presents with a well-defined rounded homogeneous opacity in the pulmonary field [8,14]. Diagnostic difficulties have been reported previously with complex HC with atypical radiographic presentations (bronchial obstruction, semi-solid lesions and consolidation)[16]; we did not face such challenges. Hydatid disease therefore should always be considered in differential diagnosis especially in endemic regions [16]. Serologic tests have poorer sensitivity for the diagnosis of pulmonary HC compared to hepatic HC [2,13,15]. The sensitivity of a serologic test ranges from 85% to 98% for hepatic HC and 50% to 60% for lung HC [5]. We did not find any significant correlation of echinococcal antibody with both the groups.

Once the diagnosis is made, the best management option is surgery [4,15]. Operating on HC mandates single lung ventilation not only to control ventilation but also to prevent contralateral lung contamination [17,18]. The rupture of cyst leading to flooding of the airway with hydatid fluid or fragments of laminated membrane can be challenging for an anesthesiologist and can also give rise to hypersensitivity reaction. Hence, single lung ventilation offers good lung protection [18]. We operated all of our cases on single lung ventilation.

The basis of the surgical therapy is removal of the entire parasite, prevention of contamination, maximum preservation of pulmonary parenchyma, identification and closure of bronchial leaks and obliteration of the remaining cavity [19]. More radical surgical procedures may be required in complex HC due to significant pleural thickening and parenchymal destruction [2,11]. In our study, the need for decortication and phrenotomy with repair was statistically significant in Group B (p<0.001). The higher decortication rate among Group B (17 [50%]) is explained by the fact that the higher rate of pleural complication was noted in the group. Aribas et al. observed that the need for decortication was 69.8% for the pleural complications associated with the cyst [9].

Studies have been conducted to compare perioperative morbidity and complications between open approach and video-assisted thoracoscopic surgery (VATS) with or without mini thoracotomy [19,20]. We operated all cases via open thoracotomy. The closure of bronchial openings and management of the residual cavity is important in order to prevent prolonged postoperative air leak and empyema formation [21]. Nabi et al. proposed it is difficult to close the bronchial openings in ruptured or infected cysts and found all of them had postoperative prolonged air leak due to cut-through of sutures leading to empyema formation. The addition of capitonnage had the advantage of securing unidentified air leaks during surgery[22].

Some authors have not used lobectomies in large series studies and have proposed lung resections to be unnecessary for HC [7]. In our study, only two cases in Group A and 12 cases in Group B required some form of lung resections. The reason for carrying out lung resections was the involvement of almost the whole of the lobe in simple cyst and the presence of unsalvageable parenchymal destruction among the complex cysts.

Postoperatively, the duration of ICU stay, duration of chest tube placement and hospital stay duration were longer in Group B (p<0.001). The need of readmission and interventions for postoperative complications were also higher in Group B (p<0.05). Postoperative prolonged air leak was managed with chest tube under negative suctioning of 15-20 cm H_2_0. Postoperative BPF were managed with prolonged tube thoracostomy attached to the Heimlich valve until lung expansion. Postoperative atelectasis responded well to chest physiotherapy. Wound infection was managed locally with wound care. Postoperative empyema was managed with either chest tube or needle thoracentesis. Both groups responded well to surgical management without mortality. We observed greater morbidity associated with complex HC that was also the observation in the study by Aribas et al. [9].

## Conclusions

Surgery has a favorable outcome in the management of HC of lung. Early diagnosis and surgery helps to prevent complications associated with the cyst rupture. Complications associated with complex HC not only require preoperative intervention like chest tubes but can also lead to life-threatening complications requiring emergency procedures like bronchoscopy and thoracotomy. There is also a frequent need for additional procedures during surgery in cases with complex HC along with greater risk of postoperative complications. All these are associated with prolonged hospital stay, readmissions and greater morbidity that add to the treatment costs of a patient from a resource-constraint area like Pakistan.
